# Design of moving average chart and auxiliary information based chart using extended EWMA

**DOI:** 10.1038/s41598-023-32781-4

**Published:** 2023-04-05

**Authors:** Muhammad Naveed, Muhammad Azam, Muhammad Shujaat Nawaz, Muhammad Saleem, Muhammad Aslam, Muhammad Saeed

**Affiliations:** 1 Department of Statistics, Govt Graduate College (B) Gulberg, Lahore, Pakistan; 2 Department of Statistics and Computer Science, University of Veterinary and Animal Sciences, Lahore, 54000 Pakistan; 3 Lecturer in Higher Education Department, Punjab, Pakistan; 4 Department of Industrial Engineering, Faculty of Engineering-Rabigh, King Abdulaziz University, Jeddah, 21589 Saudi Arabia; 5 Department of Statistics, Faculty of Science, King Abdulaziz University, Jeddah, 21551 Saudi Arabia; 6 Department of Statistics, Minhaj University, Lahore, 54770 Pakistan

**Keywords:** Engineering, Mathematics and computing

## Abstract

The control chart is the most valuable tool in the manufacturing process to track the output process in the industries. Quality specialists always want a visual framework that recognizes sustainable improvements in the monitoring processes. The efficiency of a control chart is increased by utilizing a memory-based estimator or by using any extra information relevant to the key variable. In this study, we present Extended EWMA (EEWMA) and EWMA based monitoring charts for observing the process location using moving average (MA) statistic under two different situations, i.e., when some extra information is known and unknown. We also propose an EEWMA control chart using Auxiliary Information. The output of these charts is evaluated and contrasted to the various existing charts on the basis of average run length (ARL). The comparison indicates that the proposed charts outperform rivals in identifying all types of shifts in the process location parameter. The implementation of these plans is also rendered to incorporate them in a practical situation.

## Introduction

Statistical Process Control (SPC) is a widely employed quality monitoring mechanism in different industries to track and optimize the production process. The control charts are usually classified into two main types, memoryless and memory-based charts. A memoryless chart is referred to as a Shewhart-type control chart; they only deal with the most current observation of the process and disregard any past results. The key weakness in the memoryless charting system is the less effectuality in tracing the minor changes in process parameters. Whereas, a memory-based control chart uses current as well as previous information in the sample to observe the process parameter. Memory-type charts; the cumulative sum (CUSUM) control chart suggested by Ref.^1^ and the exponentially weighted moving average (EWMA) control chart suggested by Ref.^2^ are better performed than the Shewhart-type control chart in searching out the minor variations in process parameter. These charts are more delicate to identify the minor shifts in the process parameter with a magnitude of 1.5σ or less relative to the Shewhart chart^3^. Another memory-type control chart known as a moving average (MA) based control chart alternates to CUSUM and EWMA charting structure is used to capture smaller variations in process parameters^3^. MA charting structure uses both types of information i.e., current as well as past information that’s why this structure is more powerful than the Shewhart-type control chart. In literature, we have seen so many extensions and modifications in memory type estimators to improve the competency of the proposed idea like^4^ proposed a control chart by mixing EWMA and CUSUM statistic, the resulting control chart based on mixed statistic is more efficient in searching the smaller shifts in process parameter than the individual charts. Later on Ref.^5^ suggested another mix type estimator using CUSUM and EWMA statistic for effectively watching the process location. Reference^6^ suggested the mixture of the generally weighted moving average (GWMA)‐CUSUM chart as well as its reverse order CUSUM‐GWMA chart to improve the detection competency compared with existing charts. Reference^7^ presented a control chart based on the combination of generally weighted moving average statistic and CUSUM statistic for searching out the process mean more efficiently after that Ref.^8^ proposed a control chart for monitoring the process dispersion using the combined effect of generally weighted moving average (GWMA) statistic and CUSUM statistic. Reference^9^ proposed the Extended EWMA (EEWMA) based control chart and showed much better results when compares to the competitor charts. After that Ref.^10^ constructed an EEWMA based control chart using Multiple dependent state sampling. Later on Ref.^11^ presented a repetitive sampling based control chart and showed outstanding results in terms of smaller ARL. Reference^12^ presented a mixed control chart using EWMA statistic and progressive mean for monitoring the process mean. Recently Ref.^13^ proposed the EEWMA based control chart for monitoring the shape parameter of skewed distribution. Reference^14^ presented the mixture of the GWMAV-CUSUM chart and its reverse order CUSUM-GWMAV chart to improve the searching capabilities of the GWMAV chart.

MA-based control charts have also attracted the researcher due to their effectiveness. Many researchers have developed control charts using MA statistic^15^ suggested an MA-based control chart on Banerjee and Rahim’s model. Reference^16^ presented an economic model using MA statistic and showed a better performance as compared to Shewhart-type control chart. Reference^17^ suggested a reverse MA chart for the autocorrelated process. Reference^18^ constructed a control chart based on MA statistic for watching the process fraction non-conforming after that Ref.^19^ developed a double MA charting structure for monitoring the process mean effectively. More work using MA statistic can be seen in control chart^20–23^. Recently Ref.^24^ suggested a new double MA chart. The authors discussed that the variance expression of the double MA statistic proposed by Ref.^19^ is not correct. The authors proposed the new variance expression for the double MA chart and showed better performance than the competitor chart. Recently Ref.^25^ proposed the MA based control chart using EWMA statistic for improving the competency of the proposed idea. Moving averages was first introduced in 1965^26^ and it has become very popular in recent decades as it is being used in almost all kinds of time series data. Moving averages used in medical laboratories^26^, inflation forecast^27^, river flows^28^, sales^29^, stock market predictions^30^ and many more. Reference^25^ applied MA-EWMA control chart for river data and found it to be more effective than EWMA and MA charts.

The auxiliary information is commonly used in survey sampling to design and estimate the unknown population parameter(s). Several estimators like classical ratio, product, and regression estimators are based on auxiliary information. These types of estimators needed information not only from the variable under study but also considered information from one or more associated supporting variable(s). In that case, these estimators are more accurate as compared to those estimators that only needed information under key variables. Due to the effectiveness of auxiliary information, many researchers have motivated and developed control charts using auxiliary information to ameliorate the competency of the proposed idea Ref.^31^ proposed Shewhart type estimator using auxiliary information to monitor the process mean. After that Ref.^32^ suggested another auxiliary information based control chart for searching out the process dispersion. They showed that both the proposed chart perform better than the competitor chart. Reference^33^ presented the control chart using the mixture of regression estimator (RE) and EWMA statistic. The resulting estimator performs eminent in searching out the smaller variation in the process mean. Reference^34^ proposed dual auxiliary information -based control chart using a rank set sampling scheme and proved that the newly suggested chart performed better than the competitor chart. Reference^35^ constructed a synthetic mean control chart using auxiliary information to improve the execution of synthetic mean chart. Reference^36^ developed an EWMA control chart using auxiliary information for monitoring the process mean and dispersion jointly. Reference^37^ suggested a control chart using two parametric ratio estimators to enhance the competency of the proposed idea. After that Reference^38^ offered joint monitoring charting structure of mean and coefficient of variation using with and without auxiliary information. Again Refs.^39,38^ used the auxiliary information in hybrid EWMA statistic and showed that the proposed charting structure identifies the smaller variation in the process mean more quickly. More work for the development of a control chart using auxiliary information can be seen in Refs.^40–43^. Recently Reference^12^ developed a control chart by combining the regression estimator and progressive mean and showed that the presented chart is more potent than the competitor chart. Reference^44^ developed a control chart using the combination of RE and modified EWMA statistic. The findings of the presented idea are more effective in searching out the minor variation in process location. Reference^45^ proposed control chart by utilizing a mixture of RE and MA statistic.

Reference^46^ presented that a stable auxiliary variable cannot be obtained in all situations and if the auxiliary variable shifts, the results are very much misleading. Reference^47^ proposed a control chart for a study variable that is related to an auxiliary variable. They proposed auxiliary information based (AIB) control chart when the auxiliary variable is stable and proposed modified-AIB control chart when auxiliary variable is not stable.

Reference^9^ proposed an extended EWMA (EEWMA) statistic that was found to be efficient in terms of average run length. By exploring the literature and according to the best of our knowledge, there is no work on MA and RE control charts using EEWMA statistic. The existing control charts did not incorporate auxiliary information that is linked to the main study variable. In this paper, we will present the design of MA and RE using EEWMA statistic. Motivated by the combined use of auxiliary information and memory-type control chart, we will develop various types of EEWMA control charts using auxiliary information. We will also propose auxiliary information based EWMA control chart using MA statistic. The evaluation of proposed charting structure will be evaluated by computing ARL values.

## Proposed moving average based control chart using extended EWMA statistic (EEWMA-MA control chart)

Let us assume that the subgroup average ${\overline{Y} }_{1},{\overline{Y} }_{2},{\overline{Y} }_{3}, {\overline{Y} }_{4},\dots ,$ are obtained where ${\overline{Y} }_{j}=\left({Y}_{j1}+{Y}_{j2}+{Y}_{j3}+\dots +{Y}_{jn}\right)/n$ is the $j$th subgroup and $n$ is the subgroup size. Here we presume that ${Y}_{jk}\sim N\left(\mu , {\sigma }^{2}\right)$ for $j={1,2},\dots , {\text{ and }} k={1,2},3,\dots ,n.$ The MA statistic of span $z$ at time $j$ calculated from subgroup averages ${\overline{Y} }_{1},{\overline{Y} }_{2},{\overline{Y} }_{3}, {\overline{Y} }_{4},\dots ,$ is defined as^3^

1 $${MA}_{j}=\frac{{\overline{Y} }_{j}+{\overline{Y} }_{j-1}+\dots +{\overline{Y} }_{j-z+1}}{z}$$

For $j\ge z$. for period $j<z$, we do not have $z$ a subgroup average to calculate the value of MA of width $z$. For these periods, the average of all subgroups up to the period $j$ defines the MA. Under the in-control process, the mean and variance for $j\ge z$ of ${MA}_{j}$ are

2 $$E\left({MA}_{j}\right)={\mu }_{0}$$

3 $$Var\left({MA}_{j}\right)=\frac{{\sigma }^{2}}{nz}$$

where ${\mu }_{0}$ denotes the in-control value of the process mean.

Reference^9^ proposed a memory-based statistic named extended EWMA statistic is defined as

4 $${EEWMA}_{j}= {\lambda }_{1}{\overline{Y} }_{j}-{\lambda }_{2}{\overline{Y} }_{j-1}+\left(1-{\lambda }_{1}+{\lambda }_{2}\right){EEWMA}_{j-1}$$

here ${\lambda }_{1} {\text{ and }} {\lambda }_{2}$ are smoothing constants with the condition that $0<{\lambda }_{1}\le 1 {\text{ and }} {0\le \lambda }_{2}< {\lambda }_{1},$ moreover the sum of weights is one and ${\lambda }_{1}>{\lambda }_{2}.$ The mean and variance of EEWMA statistic are

5 $$E\left({EEWMA}_{j}\right)={\mu }_{0}$$

6 $$Var\left({EEWMA}_{j}\right)=\frac{{\sigma }^{2}}{n}\left[\left({{\lambda }_{1}}^{2}+{{\lambda }_{2}}^{2}\right)\left\{\frac{1-{\gamma }^{2j}}{1-{\gamma }^{2}}\right\}-2\gamma {\lambda }_{1}{\lambda }_{2}\left\{\frac{1-{\gamma }^{2j-2}}{1-{\gamma }^{2}}\right\}\right]$$

where $\gamma =\left(1-{\lambda }_{1}+{\lambda }_{2}\right)$ and ${\mu }_{0}, {\sigma }^{2}$ indicates the target mean and variance of ${Y}_{j}$.

Now the proposed EEWMA based MA statistic denoted by *EWMA*-*MA* is defined as

7 $${EEWMA{\text{-}}MA}_{j}= {\lambda }_{1}{MA}_{j}-{\lambda }_{2}{MA}_{j}+\left(1-{\lambda }_{1}+{\lambda }_{2}\right){EEWMA}_{j-1}$$

The mean and variance of ${EEWMA{\text{-}}MA}_{j}$ statistic are

8 $$E\left({EEWMA{\text{-}}MA}_{j}\right)={\mu }_{0}$$

9 $$Var\left({EEWMA{\text{-}}MA}_{j}\right)=\frac{{\sigma }^{2}}{nz}\left[\left({{\lambda }_{1}}^{2}+{{\lambda }_{2}}^{2}\right)\left\{\frac{1-{\gamma }^{2j}}{1-{\gamma }^{2}}\right\}-2\gamma {\lambda }_{1}{\lambda }_{2}\left\{\frac{1-{\gamma }^{2j-2}}{1-{\gamma }^{2}}\right\}\right]$$

control limits of ${EEWMA{\text{-}}MA}_{j}$ statistic for $j\ge z$ are

10 $$UCL/LCL={\mu }_{0}\pm k\sqrt{\frac{{\sigma }^{2}}{nz}\left[\left({{\lambda }_{1}}^{2}+{{\lambda }_{2}}^{2}\right)\left\{\frac{1-{\gamma }^{2j}}{1-{\gamma }^{2}}\right\}-2\gamma {\lambda }_{1}{\lambda }_{2}\left\{\frac{1-{\gamma }^{2j-2}}{1-{\gamma }^{2}}\right\}\right]}$$

where $k$ is the control chart constant. The process is declared to be in-control (IC) if charting statistic falls within the limits otherwise deemed as out-of-control (OOC).

## Designing of other proposed charts using auxiliary information

Now we suppose that ${Y}_{j}$ be the study variable and is correlated with auxiliary information ${X}_{j}$. Also, we assume $\left({Y}_{j},{X}_{j}\right)$ follow the bivariate Normal distribution with mean $\left({\mu }_{Y,}{\mu }_{X}\right) , variance \left({\sigma }_{Y}^{2},{\sigma }_{X}^{2}\right)$ and the correlation between $Y and X$ is ${\rho }_{YX}$. The Regression Estimator (RE) of the population mean ${\mu }_{Y}$^48^ is

11 $${R}_{j}=\overline{{Y }_{j}}+ {\rho }_{YX}\frac{{\sigma }_{Y}}{{\sigma }_{X}}\left({\mu }_{X}-\overline{{X }_{j}}\right)$$

The mean and variance of ${R}_{j}$ are given as

12 $$E\left({R}_{j}\right){=\mu }_{0}$$

13 $$var\left({R}_{j}\right)=\frac{{\sigma }_{Y}^{2}}{n}\left(1-{\rho }_{YX}^{2}\right)$$

Now the auxiliary information based MA statistic proposed by Ref.^45^ is given as

14 $${REMA}_{j}=\frac{{R}_{j}+{R}_{j-1}+\dots +{R}_{j-z+1}}{z}$$

The mean and variance of ${REMA}_{j}$ for $j\ge z$ under in-control process is

15 $$E\left({REMA}_{j}\right){=\mu }_{0}$$

16 $$var\left({REMA}_{j}\right)=\frac{{\sigma }_{Y}^{2}}{nz}\left(1-{\rho }_{YX}^{2}\right)$$

Now the final structure of EWMA based control chart using MA statistic under the condition of auxiliary information denoted by ${EWMA{\text{-}}REMA}_{j}$ is defined as

17 $${EWMA{\text{-}}REMA}_{j}=\lambda {REMA}_{j}+\left(1-\lambda \right){EWMA{\text{-}}REMA}_{j-1}$$

The mean and variance of the newly proposed estimator when the process is in-control are given as

18 $$E\left({EWMA{\text{-}}REMA}_{j}\right){=\mu }_{0}$$

19 $$VAR\left({EWMA{\text{-}}REMA}_{j}\right)={\sigma }_{{ERMA}_{j}}^{2}=\frac{{\sigma }_{Y}^{2}}{nz}\left(1-{\rho }_{YX}^{2}\right)\left[\frac{\lambda }{2-\lambda }\left\{1-{\left(1-\lambda \right)}^{2j}\right\}\right]$$

Also, the final form of EEWMA based MA statistic using auxiliary information denoted by EEWMA-REMA is defined as

20 $${EEWMA{\text{-}}REMA}_{j}={\lambda }_{1}{REMA}_{j}-{\lambda }_{2}{REMA}_{j-1}+\left(1-{\lambda }_{1}+{\lambda }_{2}\right){EEWMA{\text{-}}REMA}_{j-1}$$

The mean and variance of ${EEWMA{\text{-}}REMA}_{j}$ statistic when the process is in-control is given as

21 $$E\left({EEWMA{\text{-}}REMA}_{j}\right){=\mu }_{0}$$

22 $$VAR\left({EEWMA{\text{-}}REMA}_{j}\right)={\sigma }_{{EEWMA{\text{-}}REMA}_{j}}^{2}=\frac{{\sigma }_{Y}^{2}}{nz}\left(1-{\rho }_{YX}^{2}\right)\left[\begin{array}{c}\left({{\lambda }_{1}}^{2}+{{\lambda }_{2}}^{2}\right)\left\{\frac{1-{\gamma }^{2j}}{1-{\gamma }^{2}}\right\}-\\ 2\gamma {\lambda }_{1}{\lambda }_{2}\left\{\frac{1-{\gamma }^{2j-2}}{1-{\gamma }^{2}}\right\}\end{array}\right]$$

The control limits of the auxiliary information based proposed chart are given as

23 $$UCL/{LCL}_{{EWMA{\text{-}}REMA}_{j}}{=\mu }_{0} \pm k\sqrt{\frac{{\sigma }_{Y}^{2}}{nz}\left(1-{\rho }_{YX}^{2}\right)\left[\frac{\lambda }{2-\lambda }\left\{1-{\left(1-\lambda \right)}^{2j}\right\}\right]}$$

24 $$UCL/{LCL}_{{EEWMA{\text{-}}REMA}_{j}}{=\mu }_{0} \pm k\sqrt{\frac{{\sigma }_{Y}^{2}}{nz}\left(1-{\rho }_{YX}^{2}\right)\left[\begin{array}{c}\left({{\lambda }_{1}}^{2}+{{\lambda }_{2}}^{2}\right)\left\{\frac{1-{\gamma }^{2j}}{1-{\gamma }^{2}}\right\}-\\ 2\gamma {\lambda }_{1}{\lambda }_{2}\left\{\frac{1-{\gamma }^{2j-2}}{1-{\gamma }^{2}}\right\}\end{array}\right]}$$

where $k$ is the control chart coefficient. The proposed charts are the extension of the various existing charts like, If we put $z=1$ in $EWMA{\text{-}}REMA$ control chart, the resulting chart is converted to the Ref.^33^. If we put $\lambda =1$ in $EWMA{\text{-}}REMA$ control chart, the presented chart is converted to Ref.^45^. The suggested chart $EWMA{\text{-}}REMA$ is transformed to the MA based control chart if ${\rho }_{YX}=0 and \lambda =1.$ The recommended chart $EWMA{\text{-}}REMA$ is converted to Shewhart control chart if we put ${\rho }_{YX}=0 , \lambda =1 and z=1.$ The proposed $EWMA{\text{-}}REMA$ control chart is converted to Ref.^25^ if we put ${\rho }_{YX}=0.$

It is important to note that if we put $z=1$ the proposed *EEWMA-REMA* control chart is converted to another proposed chart which a mixture of auxiliary information and EEWMA statistic denoted by *EEWMA-RE*. The control limits for *EEWMA-RE* statistic is given as

25 $$UCL/{LCL}_{EEWMA{\text{-}}RE }{=\mu }_{0} \pm k\sqrt{\frac{{\sigma }_{YX}^{2}}{n}\left(1-{\rho }_{YX}^{2}\right)\left[\left({{\lambda }_{1}}^{2}+{{\lambda }_{2}}^{2}\right)\left\{\frac{1-{\gamma }^{2j}}{1-{\gamma }^{2}}\right\}-2\gamma {\lambda }_{1}{\lambda }_{2}\left\{\frac{1-{\gamma }^{\left(2j-2\right)}}{1-{\gamma }^{2}}\right\}\right]}$$

The suggested $EEWMA{\text{-}}RE$ based charting structure is converted to the Ref.^9^ if we put ${\rho }_{YX}=0.$ The presented charting schemes are considered to be IC if the value of plotting statistic lies between the limits. Contrarily, it is declared as OOC. The output of the presented charting structure is evaluated by measuring the ARL. The ARL is known to be the most widely used metric for assessing and analyzing the charting structure^3^. The ARL is defined as the average number of subgroups before the OOC signal is displayed. The IC ARLs of the presented charting structure have been calculated for various combinations of the parameters and the control constant $k$ has been determined at which the IC ARL equals the stated value. After that, we tabulate the OOC ARLs for different combinations of other parameters.

In Tables, the ${ARL}_{0}$ is set to 370, while the correlation coefficient ${\rho }_{YX}$ for the population is assumed to be known because in most of the realistic scenarios, it is known already^49^. The values of ARLs are determined using the Monte Carlo technique and the codes are written in R language.

The Monte Carlo technique is explained below.

1. Generate a random sample of size n using a bivariate normal distribution (BND) with parameters $\left(Y,X\right)\sim {\text{N}}_{2}\left({\mu }_{Y},{\mu }_{X}{1,1},{\rho }_{YX}\right)$, considering a mean shift that is $\left(Y,X\right)\sim {\text{N}}_{2}\left({\mu }_{Y}+c{\sigma }_{Y},{\mu }_{X}{1,1},{\rho }_{YX}\right)$. For the case of univariate, a sample is drawn from a standard normal distribution that is $Y\sim \text{N}\left({0,1}\right)$.
2. Select the values of the smoothing constant and $k$ according to the selected ${ARL}_{0}$ and calculate the control limits.
3. Calculate the value of the proposed estimator.
4. Plot the values of the presented estimator on the control chart.
5. Run the process until the value of the suggested estimator is out of control, count the points to determine the run length.
6. Repeat points 4–6, a very large number of times say 50,000 and calculate the ARL of the process. After that again start the process from point 4.

## Results and discussion

First of all the proposed control charts use more computations and understanding than usual Shewhart control charts, EWMA control charts, MA control charts, etc. but by using the proposed control charts, we are flexible in various conditions as we have more control parameters to specify. Setting specific control parameter values, the proposed control chart is converted to other previously presented control charts as mentioned in the previous part.

Table 1 displays the OOC ARL values (or ${ARL}_{1}$) for different span size z (= 3, 5 and 10), smoothing constants, and shift constant $c$ (between 0.0 and 2.0) when ${ARL}_{o}=370$ and sample size $n=5$ for EEWMA-MA control chart. It is found that for smaller values of smoothing constants, the execution of the presented chart is much better for larger values of the smoothing constant which indicates earlier identification in process shift. For example, when ${ARL}_{0}=370, z=3, n=5 , c=0.04 and {\lambda }_{1}=0.1, {\lambda }_{2}=0.03$ the ARL value of the presented chart is 246.79 and for the smoothing constant ${\lambda }_{1}=0.2, {\lambda }_{2}=0.07$, the value of the suggested chart is increased to 275.49. We also noticed that the recommended charting structure performed better with a larger value of span $Z$. We also see a quicker identification in the process mean for a higher value of shifted constant $c$ when all the other parametric values have remained the same. For example, when ${ARL}_{0}=370, z=10, n=5 , c=2 {\text{ and }} {\lambda }_{1}=0.1, {\lambda }_{2}=0.03$ the ARL value of the presented chart is just 1 it shows that the presented chart identified the shift in the very first sample.

**Table 1:** ARLs of *EEWMA-MA* control chart when ${ARL}_{0}=370, n=5$.

	${\lambda }_{1}=0.1, {\lambda }_{2}=0.03$			${\lambda }_{1}=0.2, {\lambda }_{2}=0.07$			${\lambda }_{1}=0.3, {\lambda }_{2}=0.15$		
	$z=3$	$z=5$	$z=10$	$z=3$	$z=5$	$z=10$	$z=3$	$z=5$	$z=10$
$k$→	4.155	5.018	6.384	4.150	4.863	5.837	3.899	4.427	5.174
c	$ARL (SDRL)$	$ARL (SDRL)$	$ARL (SDRL)$	$ARL (SDRL)$	$ARL (SDRL)$	$ARL (SDRL)$	$ARL (SDRL)$	$ARL (SDRL)$	$ARL (SDRL)$
0	369.85 (374.04)	370.07 (392.88)	370.11 (407.8)	370.27 (379.13)	370.11 (381.82)	369.94 (410.14)	369.95 (372.46)	370.22 (377.08)	370.28 (419.28)
0.03	279.00 (291.5)	273.39 (286.05)	267.78 (297.36)	310.06 (313.21)	308.37 (313.34)	290.30 (323.52)	318.98 (319.64)	312.60 (317.56)	305.89 (325.5)
0.04	246.79 (250.97)	238.07 (249.19)	232.97 (257.35)	275.49 (274.83)	268.50 (269.79)	255.70 (260.61)	291.29 (295.37)	277.99 (289.98)	264.58 (299.62)
0.05	207.84 (209.39)	198.54 (202.48)	194.18 (214.95)	239.04 (270.22)	230.73 (232.01)	216.38 (241.94)	252.56 (254.96)	240.29 (245.34)	226.02 (257.57)
0.06	171.44 (172.98)	165.72 (173.76)	162.53 (174.73)	203.47 (208.2)	197.04 (198.12)	179.13 (189.95)	226.74 (223.1)	208.55 (218.56)	193.59 (219.21)
0.07	146.64 (147.39)	137.98 (143.04)	132.64 (143.63)	178.16 (179.09)	171.31 (175.28)	148.56 (170.29)	193.97 (189.44)	179.78 (181.76)	164.01 (185.59)
0.08	121.18 (116.87)	117.17 (117.91)	111.10 (121.98)	152.23 (150.62)	145.46 (149.44)	131.38 (147.57)	165.95 (166.06)	153.88 (156.06)	137.59 (154.92)
0.09	101.82 (99.38)	97.45 (98.09)	93.41 (99.11)	131.18 (129.85)	122.38 (123.57)	109.34 (124.62)	144.10 (144.21)	132.16 (134.15)	101.78 (109.87)
0.10	87.99 (86.99)	82.70 (85.12)	79.24 (82.29)	113.79 (113.32)	106.77 (107.53)	93.50 (99.07)	123.36 (122.03)	115.29 (116.29)	99.81 (110.86)
0.12	63.86 (61.09)	60.57 (59.67)	57.59 (56.49)	84.30 (83.79)	79.03 (77.72)	66.96 (72.06)	95.45 (94.67)	86.42 (85.43)	73.19 (82.05)
0.15	45.36 (41.17)	43.04 (40.86)	39.41 (39.68)	58.60 (57.65)	52.91 (52.14)	45.34 (47.37)	65.66 (64.77)	58.65 (57.21)	48.43 (51.39)
0.17	36.09 (30.75)	34.23 (33.13)	31.98 (30.89)	45.72 (41.64)	42.97 (41.56)	36.49 (37.47)	51.86 (48.78)	45.86 (44.51)	38.87 (40.65)
0.20	27.27 (30)	24.77 (24.78)	22.87 (24.07)	33.68 (29.73)	30.93 (29.13)	26.77 (26.43)	36.86 (32.89)	33.85 (31.86)	26.85 (28.03)
0.22	23.25 (18.6)	21.78 (17.62)	20.27 (18.14)	28.35 (24.24)	26.01 (23.4)	21.74 (21.28)	30.90 (26.93)	27.90 (25.68)	22.80 (23.1)
0.25	19.15 (14.18)	17.18 (13.33)	16.76 (14.18)	22.57 (18.64)	20.44 (17.98)	16.94 (17.59)	23.95 (19.99)	20.95 (19.06)	17.87 (18.9)
1.00	1.89 (1.1)	1.66 (1.08)	1.58 (0.94)	1.89 (1.13)	1.71 (1.09)	1.60 (0.9)	1.91 (1.14)	1.72 (1.1)	1.40 (0.77)
2.00	1.00 (0.1)	1.00 (0.09)	1.00 (0.09)	1.00 (0.11)	1.00 (0.1)	1.00 (0.09)	1.00 (0.11)	1.00 (0.1)	1.00 (0.09)

Table 2 presents the ARLs of the *EEWMA-RE*, auxiliary information based EEWMA charting structure. We see that the performance of the presented charting structure is increased in the form of early identification of process parameters by introducing the role of auxiliary information. We notice that the values of ARL decreased very sharply for larger values of $\rho$. Tables 3, 4, 5, 6, 7 and 8 show the values of ARLs based on EWMA-REMA and EEWMA-REMA control charts. We notice that for a larger value of MA span, the detection ability of the presented chat has increased. We also observe the outstanding performance of suggested charts for a larger value of $\rho$.

**Table 2:** ARLs of *EEWMA-RE* control chart when ${ARL}_{0}=370, n=5$.

	$\rho =0.50$			$\rho =0.90$		
	${\lambda }_{1}=0.1$ ${\lambda }_{2}=0.03$	${\lambda }_{1}=0.2$ ${\lambda }_{2}=0.07$	${\lambda }_{1}=0.3$ ${\lambda }_{2}=0.15$	${\lambda }_{1}=0.1$ ${\lambda }_{2}=0.03$	${\lambda }_{1}=0.2$ ${\lambda }_{2}=0.07$	${\lambda }_{1}=0.3$ ${\lambda }_{2}=0.15$
k>>>	2.719	2.887	2.956	2.719	2.887	5.902
C	$ARL$ $(SDRL)$	$ARL (SDRL)$	$ARL$ $(SDRL)$	$ARL$ $(SDRL)$	$ARL$ $(SDRL)$	$ARL$ $(SDRL)$
0	370.11 (366.66)	370.01 (369.59)	369.97 (369.13)	370.00 (369.18)	370.12 (372.86)	370.11 (367.84)
0.03	271.80 (274.68)	306.48 (307.46)	314.06 (307.13)	149.22 (144.11)	190.55 (185.67)	215.94 (211.98)
0.04	227.85 (224.76)	257.08 (246.54)	279.52 (276.44)	99.74 (93.64)	137.48 (133.85)	162.42 (158.02)
0.05	184.12 (183.12)	229.53 (224.48)	249.60 (240.8)	72.77 (67.57)	100.37 (93.95)	117.92 (109.22)
0.06	152.42 (144.36)	189.90 (187.93)	212.97 (205.16)	53.90 (47.04)	73.43 (69)	94.36 (86.16)
0.07	121.39 (115.53)	156.32 (152.36)	182.39 (177.44)	42.09 (35.9)	56.04 (50.06)	68.36 (63.41)
0.08	102.61 (98.19)	135.68 (133.7)	163.47 (159.62)	33.03 (26.96)	45.09 (38.1)	54.76 (48.54)
0.09	88.36 (81.83)	114.82 (108.9)	136.74 (131.9)	26.96 (21.1)	36.56 (30.03)	43.74 (36.82)
0.10	70.80 (66.16)	98.03 (96.6)	120.90 (117.25)	20.99 (17.13)	29.03 (23.16)	35.72 (31.21)
0.12	54.01 (48.93)	73.55 (67.3)	88.23 (82.81)	16.68 (11.16)	21.17 (16.18)	25.06 (20.21)
0.15	36.90 (31.04)	49.62 (43.69)	60.62 (54.68)	10.82 (5.2)	13.19 (9.2)	16.04 (11.11)
0.17	29.14 (23.73)	37.66 (33.13)	47.71 (42.22)	9.38 (5.97)	11.09 (7.39)	13.19 (8.14)
0.20	21.99 (17.24)	28.66 (23.05)	36.45 (30.9)	7.22 (4.5)	8.21 (5.21)	9.99 (6.17)
0.22	19.01 (13.24)	24.57 (19)	28.74 (24.08)	5.97 (3.19)	7.10 (4.11)	8.45 (4.95)
0.25	15.14 (11.16)	19.00 (14.15)	22.89 (17.92)	4.98 (2.92)	5.81 (3.33)	6.89 (3.77)
1.00	1.76 (0.8)	1.85 (0.86)	1.92 (0.9)	1.00 (0)	1.01 (0.1)	1.01 (0.11)
2.00	1.00 (0.07)	1.01 (0.09)	1.01 (0.1)	1.00 (0)	1.00 (0)	1.00 (0)

**Table 3:** ARLs of EWMA-REMA control chart when ${ARL}_{0}=370, n=5,\lambda =0.10$.

	$k=4.275$		$k=5.175$		$k=6.463$	
	$z=3$		$z=5$		$z=10$	
	$\rho =0.50$	$\rho =0.90$	$\rho =0.50$	$\rho =0.90$	$\rho =0.50$	$\rho =0.90$
C	$ARL (SDRL)$	$ARL$ $(SDRL)$	$ARL$ $(SDRL)$	$ARL$ $(SDRL)$	$ARL$ $(SDRL)$	$ARL$ $(SDRL)$
0	370.12 (384.9)	369.99 (377.75)	370.08 (391.81)	369.89 (380.93)	370.01 (409.09)	370.02 (411.17)
0.03	275.84 (282.7)	160.34 (169.35)	274.23 (290.34)	156.18 (162.45)	266.32 (293.18)	150.66 (167.47)
0.04	235.93 (239.91)	111.88 (108.03)	231.34 (251.43)	110.22 (113.54)	221.45 (248.57)	101.07 (110.08)
0.05	197.13 (201.33)	77.37 (74.2)	195.34 (204.54)	76.72 (73.96)	182.68 (200.71)	71.22 (75.44)
0.06	161.31 (161.53)	59.32 (56.06)	157.45 (165.34)	56.00 (54.99)	148.20 (164.86)	51.20 (52.1)
0.07	132.45 (130.66)	44.73 (41.87)	127.23 (128.87)	43.15 (39.88)	121.84 (131.44)	38.72 (38.91)
0.08	111.67 (110.78)	34.84 (31.16)	108.12 (111.23)	34.00 (31.93)	102.50 (111.18)	30.25 (30.39)
0.09	94.20 (86.79)	28.14 (24.49)	90.89 (89.45)	27.16 (23.87)	84.75 (88.72)	24.37 (23.27)
0.10	78.72 (75.96)	22.87 (19.24)	75.95 (73.28)	22.05 (18.91)	71.52 (77.42)	19.98 (18.52)
0.12	57.83 (55.06)	16.46 (13.08)	56.12 (55.65)	15.93 (12.06)	50.68 (51.17)	14.36 (12.91)
0.15	40.14 (35.05)	11.30 (5.42)	37.34 (35.54)	10.75 (8.26)	33.83 (33.57)	9.73 (8.03)
0.17	31.47 (26.3)	8.99 (6.37)	29.41 (26.18)	8.58 (6.54)	26.99 (25.37)	7.83 (6.41)
0.20	22.95 (19.13)	6.94 (4.8)	21.75 (18.83)	6.67 (4.71)	19.66 (18.58)	5.92 (4.84)
0.22	19.50 (15.62)	5.82 (3.84)	18.65 (15.51)	5.64 (3.99)	16.76 (14.61)	5.04 (3.93)
0.25	15.56 (11.99)	4.74 (3.02)	14.76 (12.03)	4.56 (3.17)	13.02 (11.34)	3.98 (3.12)
1.00	1.66 (0.85)	1.00 (0.06)	1.55 (0.82)	1.00 (0.05)	1.38 (0.7)	1.00 (0.03)
2.00	1.00 (0.05)	1.00 (0)	1.00 (0.05)	1.00 (0)	1.00 (0.03)	1.00 (0)

**Table 4:** ARLs of EWMA-REMA control chart when ${ARL}_{0}=370, n=5,\lambda =0.20$.

	$k=4.306$		$k=5.02$		$k=5.903$	
	$z=3$		$z=5$		$z=10$	
	$\rho =0.50$	$\rho =0.90$	$\rho =0.50$	$\rho =0.90$	$\rho =0.50$	$\rho =0.90$
c	$ARL$ $(SDRL)$	$ARL$ $(SDRL)$	$ARL$ $(SDRL)$	$ARL$ $(SDRL)$	$ARL$ $(SDRL)$	$ARL$ $(SDRL)$
0	370.02 (381.25)	370.01 (378.47)	370.04 (387.5)	369.88 (383.8)	369.91 (383.91)	374.34 (389.53)
0.03	303.31 (309.01)	200.59 (201.99)	297.54 (305.07)	185.14 (194.46)	288.38 (383.91)	172.29 (197.27)
0.04	269.96 (273.71)	146.19 (147.97)	253.70 (268.5)	139.05 (145.16)	244.31 (281.46)	119.12 (136.76)
0.05	233.09 (239.97)	107.04 (108.01)	222.99 (232.72)	100.02 (102.27)	202.60 (231.38)	86.34 (97.56)
0.06	201.01 (206.24)	79.19 (78.09)	189.18 (203.42)	71.66 (73.56)	177.74 (204.53)	61.23 (69.98)
0.07	169.95 (173.7)	60.43 (57.67)	157.17 (166.12)	54.78 (55.51)	145.24 (162.45)	45.91 (51.53)
0.08	148.09 (148.03)	46.72 (46.28)	134.62 (144.56)	41.82 (41.3)	119.51 (135.83)	34.48 (37.76)
0.09	125.49 (126.73)	36.75 (34.09)	116.42 (121.93)	33.58 (32.52)	99.93 (114.81)	27.23 (29.68)
0.10	104.13 (105.76)	29.84 (27.07)	96.54 (101.73)	26.88 (25.58)	84.93 (96.91)	22.32 (23.15)
0.12	80.15 (80.09)	20.65 (18.66)	70.80 (74.11)	18.27 (17.16)	60.61 (67.37)	15.12 (15.25)
0.15	52.07 (51.62)	13.17 (10.69)	45.95 (46.59)	11.56 (9.78)	39.81 (43.91)	9.61 (9.18)
0.17	41.15 (39.93)	10.19 (8)	36.01 (35.79)	9.03 (7.3)	30.74 (33.13)	7.55 (7.03)
0.20	29.19 (26.96)	7.36 (5.4)	26.67 (25.17)	6.69 (5.25)	21.33 (22.3)	5.43 (4.83)
0.22	24.79 (22.74)	6.19 (4.35)	21.80 (20.37)	5.64 (4.33)	17.91 (18.81)	4.57 (4.07)
0.25	18.66 (16.31)	4.94 (3.38)	16.71 (15.23)	4.48 (3.24)	13.73 (13.76)	3.59 (3.03)
1.00	1.65 (0.85)	1.00 (0.06)	1.51 (0.79)	1.00 (0.03)	1.30 (0.61)	1 (0.02)
2.00	1.00 (0.06)	1.00 (0)	1.00 (0.03)	1.00 (0)	1.00 (0)	1 (0)

**Table 5:** ARLs of EWMA-REMA control chart when ${ARL}_{0}=370, n=5,\lambda =0.30$.

	$k=4.205$		$k=4.779$		$k=5.344$	
	$z=3$		$z=5$		$z=10$	
	$\rho =0.50$	$\rho =0.90$	$\rho =0.50$	$\rho =0.90$	$\rho =0.50$	$\rho =0.90$
c	$ARL$ $(SDRL)$	$ARL$ $(SDRL)$	$ARL$ $(SDRL)$	$ARL$ $(SDRL)$	$ARL$ $(SDRL)$	$ARL$ $(SDRL)$
0	369.92 (376.32)	370.01 (376.28)	369.94 (394.5)	370.18 (387.61)	370.17 (447.07)	370.14 (415.14)
0.03	318.32 (319.36)	221.62 (226.33)	302.98 (324.6)	218.87 (235.18)	306.05 (362.41)	188.24 (225.07)
0.04	288.09 (289.08)	170.12 (176.7)	280.17 (301.12)	159.01 (166.15)	249.07 (294.03)	129.24 (155.21)
0.05	255.90 (260.07)	124.49 (127.19)	244.96 (265.83)	119.35 (124.19)	223.78 (265.2)	94.29 (111.22)
0.06	221.22 (219.48)	97.36 (98.66)	213.28 (225.1)	87.09 (92.44)	188.62 (228.23)	66.89 (79.73)
0.07	194.94 (197.77)	73.13 (74.34)	184.00 (197.26)	66.16 (68.81)	151.01 (183.1)	48.61 (58.77)
0.08	169.63 (173.74)	56.68 (56.43)	156.31 (163.33)	51.54 (53.82)	127.50 (153.25)	37.05 (44.06)
0.09	145.06 (149.06)	44.73 (44.24)	133.36 (141.93)	39.82 (42.59)	110.95 (133.12)	29.08 (33.7)
0.10	126.82 (129.83)	35.99 (35.29)	116.74 (123.04)	31.45 (32.03)	93.16 (108.8)	23.21 (26.07)
0.12	96.81 (98.67)	24.16 (22.15)	85.79 (90.59)	21.60 (21.32)	66.73 (78.74)	15.42 (17.39)
0.15	64.95 (63.95)	14.48 (12.73)	55.35 (57.16)	13.10 (12.38)	42.87 (50.8)	9.23 (9.9)
0.17	49.91 (49.15)	11.27 (9.79)	44.49 (46.47)	9.69 (8.93)	32.91 (38.18)	7.04 (7.27)
0.20	35.72 (34.11)	7.97 (8.43)	30.96 (31.79)	7.00 (6.02)	22.72 (26.6)	4.96 (5.02)
0.22	28.96 (28.18)	6.52 (5.11)	25.33 (25.45)	5.71 (4.73)	18.30 (20.75)	4.13 (3.97)
0.25	21.86 (20.3)	5.06 (3.64)	18.79 (18.75)	4.38 (3.5)	13.97 (15.45)	3.28 (2.88)
1.00	1.61 (0.85)	1.00 (0.05)	1.43 (0.72)	1.00 (0.04)	1.21 (0.51)	1.00 (0.02)
2.00	1.00 (0.06)	1.00 (0)	1.00 (0.03)	1.00 (0)	1.00 (0.01)	1.00 (0)

**Table 6:** ARLs of EEWMA-REMA control chart ${ARL}_{0}=370, n=5,{\lambda }_{1}=0.1,{\lambda }_{2}=0.03$.

	$k=4.154$		$k=5.018$		$k=6.384$	
	$z=3$		$z=5$		$z=10$	
	$\rho =0.50$	$\rho =0.90$	$\rho =0.50$	$\rho =0.90$	$\rho =0.50$	$\rho =0.90$
c	$ARL$ $(SDRL)$	$ARL$ $(SDRL)$	$ARL$ $(SDRL)$	$ARL$ $(SDRL)$	$ARL$ $(SDRL)$	$ARL$ $(SDRL)$
0	370.11 (382.29)	369.95 (380.77)	370.12 (387.24)	369.87 (374.16)	370.09 (414.62)	370.10 (411.99)
0.03	260.36 (264.17)	144.80 (142.63)	269.22 (282.4)	140.56 (142.53)	258.25 (289.09)	139.45 (151.07)
0.04	212.97 (216.03)	100.58 (97.52)	217.79 (220.79)	96.55 (95.78)	207.81 (225.4)	93.04 (95.14)
0.05	172.87 (174.17)	71.36 (66.38)	175.77 (183.75)	68.18 (64.33)	165.28 (178.23)	66.03 (65.56)
0.06	144.57 (145.65)	51.74 (46.12)	144.83 (147.82)	51.21 (44.82)	134.62 (145.8)	46.98 (46.2)
0.07	115.56 (114.42)	40.23 (35.1)	117.99 (120.08)	38.91 (36.32)	112.11 (119.75)	37.04 (35.02)
0.08	95.90 (92.38)	32.14 (26.93)	95.93 (94.04)	30.82 (27.92)	90.79 (96.18)	28.42 (25.94)
0.09	82.20 (77.93)	26.18 (20.93)	79.96 (77.96)	24.95 (20.64)	76.95 (78.02)	23.81 (21.15)
0.10	69.85 (64.54)	21.78 (16.86)	69.98 (67.98)	20.86 (18.77)	64.31 (63.56)	19.26 (16.68)
0.12	51.04 (45.9)	15.96 (11.95)	50.02 (46.03)	15.23 (12.9)	47.45 (45.73)	14.11 (12.85)
0.15	35.16 (29.48)	11.01 (7.7)	33.59 (29.1)	10.34 (7.04)	32.02 (29.79)	9.77 (6.43)
0.17	28.11 (22.68)	8.93 (6.11)	27.39 (23.43)	8.42 (6.38)	25.17 (22.22)	7.87 (6.19)
0.20	21.11 (16.73)	6.78 (4.5)	20.90 (16.8)	6.47 (4.44)	19.06 (16.78)	5.94 (4.67)
0.22	18.25 (13.86)	5.82 (3.74)	17.63 (13.75)	5.56 (3.6)	16.16 (13.74)	5.06 (3.95)
0.25	14.42 (10.67)	4.72 (3.01)	14.43 (11.01)	4.52 (3.03)	12.90 (10.69)	4.00 (3.11)
1.00	1.66 (0.84)	1.00 (0.06)	1.54 (0.83)	1.00 (0.05)	1.36 (0.7)	1.00 (0.03)
2.00	1.00 (0.05)	1.00 (0)	1.00 (0.04)	1.00 (0)	1.00 (0.03)	1.00 (0)

**Table 7:** ARLs of EEWMA-REMA control chart when ${ARL}_{0}=370, n=5,{\lambda }_{1}=0.2,{\lambda }_{2}=0.07$.

	$k=4.1505$		$k=4.863$		$k=5.8378$	
	$z=3$		$z=5$		$z=10$	
	$\rho =0.50$	$\rho =0.90$	$\rho =0.50$	$\rho =0.90$	$\rho =0.50$	$\rho =0.90$
c	$ARL$ $(SDRL)$	$ARL$ $(SDRL)$	$ARL$ $(SDRL)$	$ARL$ $(SDRL)$	$ARL$ $(SDRL)$	$ARL$ $(SDRL)$
0	370.22 (372.31)	370.10 (375.66)	369.89 (375.91)	370.08 (373.64)	369.95 (392.45)	370.20 (396.16)
0.03	294.63 (298.35)	179.42 (182.5)	290.22 (302.14)	178.42 (181.65)	270.15 (304.09)	157.65 (171.84)
0.04	249.65 (256.13)	130.89 (127.94)	244.86 (254.75)	124.69 (122.9)	232.60 (258.31)	109.77 (119.81)
0.05	218.99 (217.48)	91.05 (88.34)	205.21 (210.68)	86.37 (85.12)	191.24 (216.8)	78.40 (84.81)
0.06	182.29 (179.24)	67.26 (64.69)	173.87 (180.11)	62.91 (62.8)	157.32 (175.1)	55.57 (57.85)
0.07	148.78 (147.23)	52.70 (48.85)	142.21 (144.73)	47.78 (45.47)	124.27 (137.78)	41.91 (43.88)
0.08	124.95 (122.62)	39.92 (34.88)	123.69 (128.41)	37.55 (33.75)	107.92 (119.86)	32.10 (32.25)
0.09	105.61 (103.2)	31.14 (27.5)	100.31 (99.24)	30.08 (27.29)	89.42 (98.27)	26.04 (25.24)
0.10	90.21 (86.45)	26.08 (22.31)	84.53 (85.43)	24.15 (21.32)	75.08 (80.26)	20.60 (19.52)
0.12	65.46 (62.47)	18.74 (14.09)	62.43 (61.36)	17.13 (14.66)	54.72 (56.92)	14.13 (12.89)
0.15	43.40 (40.18)	12.29 (8.98)	41.41 (38.42)	11.25 (8.88)	35.90 (37.2)	9.43 (8.57)
0.17	35.37 (31.32)	9.64 (6.78)	32.61 (29.4)	8.97 (7.08)	28.10 (27.69)	7.32 (6.5)
0.20	26.28 (22.31)	7.26 (4.74)	24.01 (21.32)	6.41 (5.06)	20.22 (19.79)	5.07 (4.8)
0.22	21.24 (17.35)	6.06 (4.13)	20.05 (17.01)	5.42 (4.15)	17.01 (16.01)	4.33 (3.88)
0.25	16.99 (13.21)	4.96 (3.24)	15.81 (12.97)	4.57 (3.16)	13.25 (12.11)	3.73 (3)
1.00	1.68 (0.86)	1.00 (0.07)	1.51 (0.77)	1.00 (0.05)	1.29 (0.62)	1.00 (0)
2.00	1.00 (0.06)	1.00 (0)	1.00 (0.02)	1.00 (0)	1.00 (0)	1.00 (0)

**Table 8:** ARLs of EEWMA-REMA control chart when ${ARL}_{0}=370, n=5,{\lambda }_{1}=0.3,{\lambda }_{2}=0.15$.

	$k=3.899$		$k=4.427$		$k=5.174$	
	$z=3$		$z=5$		$z=10$	
	$\rho =0.50$	$\rho =0.90$	$\rho =0.50$	$\rho =0.90$	$\rho =0.50$	$\rho =0.90$
c	$ARL$ $(SDRL)$	$ARL$ $(SDRL)$	$ARL$ $(SDRL)$	$ARL$ $(SDRL)$	$ARL$ $(SDRL)$	$ARL$ $(SDRL)$
0	370.25 (371.45)	370.16 (374.29)	370.02 (374.04)	370.20 (374.13)	370.65 (369.45)	370.09 (387.65)
0.03	292.34 (− 301.03)	197.57 (− 197.19)	285.55 (− 305.37)	181.77 (− 187.07)	286.58 (− 320.69)	168.57 (− 188.32)
0.04	269.78 (− 272.41)	139.37 (− 137.92)	253.76 (− 261.32)	132.24 (− 133.68)	240.31 (− 270.95)	117.32 (− 131.31)
0.05	224.49 (− 226.36)	103.97 (− 99.87)	211.10 (− 213.71)	94.14 (− 95.05)	202.78 (− 233.69)	80.27 (− 85)
0.06	193.15 (− 194.51)	75.78 (− 73.41)	181.75 (− 186.12)	68.41 (− 68.31)	167.37 (− 185.71)	58.11 (− 63.47)
0.07	161.82 (− 160.58)	57.63 (− 54.54)	152.88 (− 156.38)	51.33 (− 49.92)	138.02 (− 153.24)	44.41 (− 46.87)
0.08	139.25 (− 135.07)	44.78 (− 40.16)	130.42 (− 135.53)	40.82 (− 38.57)	112.78 (− 126.64)	33.69 (− 34.78)
0.09	117.91 (− 113.42)	35.66 (− 31.72)	108.16 (− 109.23)	31.95 (− 29.27)	94.02 (− 105.18)	26.48 (− 26.62)
0.10	102.50 (− 101.35)	28.85 (− 25.14)	91.78 (− 91.27)	26.25 (− 23.99)	79.31 (− 85.8)	21.71 (− 22.18)
0.12	75.87 (− 72.13)	20.09 (− 16.16)	66.69 (− 65.18)	18.18 (− 15.61)	57.75 (− 62.09)	14.64 (− 14.18)
0.15	50.46 (− 46.79)	13.37 (− 10.03)	45.31 (− 43.04)	11.67 (− 9.35)	37.33 (− 39.21)	9.02 (− 8.55)
0.17	40.43 (− 38)	10.52 (− 7.68)	35.78 (− 32.95)	9.25 (− 7.16)	28.68 (− 29.89)	6.96 (− 6.73)
0.20	29.15 (− 22.33)	7.89 (− 5.27)	25.16 (− 22.86)	6.82 (− 5.02)	20.46 (− 20.31)	4.94 (− 4.84)
0.22	23.68 (− 20.18)	6.19 (− 4.29)	21.50 (− 18.85)	5.44 (− 4.21)	17.23 (− 16.65)	3.99 (− 3.96)
0.25	18.73 (− 15)	4.99 (− 3.35)	16.49 (− 14.03)	4.15 (− 3.22)	13.32 (− 12.57)	3.12 (− 3.03)
1.00	1.52 (− 0.92)	1.00 (− 0.04)	1.41 (− 0.77)	1.00 (− 0.03)	1.20 (− 0.59)	1.00 (− 0.02)
2.00	1.00 (− 0.05)	1.00 (0)	1.00 (− 0.03)	1.00 (0)	1.00 (− 0.01)	1.00 (0)

## Comparisons of proposed control charts with previous control charts

In this section, the proposed control charts are compared with already presented control charts in the literature.

### Proposed *EEWMA-MA* control chart verses^9^

In this section, we made a comparison between the presented chart and Ref.^9^ with the help of ARL. The presented charting structure is converted to Ref.^9^. If we set the MA span $z=1$. To get a clear picture of the recommended chart, the rest of the parametric values are the same except for the MA span for comparison purposes. From Table 9, we see that offered chart has a lower ARL value as compared to the Ref.^9^. For example, when ${ARL}_{0}=370,{\lambda }_{1}=0.1$, ${\lambda }_{2}=0.03 c=0.04$ and $z=10$ the ARL for the presented chart is 196.34 and it was 214.34 for Ref.^9^ with all other values being the same. Similarly for ${\lambda }_{1}=0.2,{\lambda }_{2}=0.07 {\text{ and }} c=0.07 {\text{ and }} z=10$ the value of ARL for the suggested chart is 151.13 and it was 191.67^9^. We see the remarkable difference between ARL, which shows the amazing performance of the suggested chart in the form of early shift detection.

**Table 9:** Comparisons of the proposed EEWMA-MA control chart versus existing charts when ${ARL}_{0}=370, n=5$.

	Proposed EEWMA-MA	^9^	^25^	Proposed EEWMA-MA	^9^	^25^
	${\lambda }_{1}=0.1$ ${\lambda }_{2}=0.03$	${\lambda }_{1}=0.1$ ${\lambda }_{2}=0.03$	$\lambda =0.10$	${\lambda }_{1}=0.2$ ${\lambda }_{2}=0.07$	${\lambda }_{1}=0.2$ ${\lambda }_{2}=0.07$	$\lambda =0.20$
	$k=6.385$	$k=2.7194$	$k=6.463$	$k=5.8378$	$k=2.8874$	$k=5.903$
	$z=10$	$z=1$	$z=10$	$z=10$	$z=1$	$z=10$
C	$ARL$ $(SDRL)$	$ARL$ $(SDRL)$	$ARL$ $(SDRL)$	$ARL$ $(SDRL)$	$ARL$ $(SDRL)$	$ARL$ $(SDRL)$
0	374.23 (412.34)	372.45 (373.43)	375.45 (406.78)	376.34 (417.23)	371.34 (374.23)	375.87 (416.56)
0.03	270.76 (300.67)	287.41 (290.12)	286.34 (331.43)	295.32 (329.12)	320.45 (320.28)	312.98 (358.12)
0.04	235.56 (260.21)	251.12 (248.12)	247.45 (278.45)	260.12 (265.12)	287.80 (290.12)	272.23 (313.42)
0.05	196.34 (217.34)	214.34 (215.67)	210.34 (234.87)	220.12 (246.12)	255.12 (252.43)	234.45 (267.12)
0.06	164.34 (176.67)	178.34 (175.34)	178.90 (200.78)	182.23 (193.23)	221.23 (220.34)	207.12 (238.18)
0.07	134.12 (145.23)	150.32 (149.34)	149.09 (161.78)	151.13 (173.23)	191.67 (185.34)	175.34 (201.23)
0.08	112.34 (123.34)	124.34 (116.34)	125.34 (136.87)	133.65 (150.12)	165.34 (161.92)	148.12 (170.32)
0.09	94.45 (100.21)	107.45 (101.61)	104.12 (111.98)	111.23 (126.78)	141.34 (138.54)	130.23 (148.98)
0.10	80.12 (83.21)	91.23 (87.12)	89.34 (94.89)	95.12 (100.78)	122.32 (117.89)	107.12 (122.67)
0.12	58.23 (57.12)	67.12 (65.78)	66.98 (71.67)	68.12 (73.31)	92.86 (86.12)	80.12 (90.32)
0.15	(39.45 (40.12)	48.21 (49.12)	45.13 (45.92)	46.12 (48.19)	64.23 (59.23)	53.23 (58.12)
0.17	32.34 (31.23)	39.21 (38.14)	35.14 (34.43)	37.12 (38.12)	51.32 (46.15)	41.23 (45.98)
0.20	23.12 (24.34)	29.21 (28.18)	27.12 (25.12)	27.23 (26.89)	38.13 (33.90)	30.12 (32.43)
0.22	20.5 (18.34)	24.65 (23.89)	22.21 (21.34)	22.12 (21.65)	31.23 (26.81)	24.98 (25.18)
0.25	16.95 (14.34)	20.12 (19.56)	18.05 (15.78)	17.23 (17.89)	25.17 (20.44)	19.23 (18.56)
1.00	1.60 (0.95)	2.11 (1.91)	1.68 (0.99)	1.63 (0.92)	2.30 (1.05)	1.53 (0.90)
2.00	1.0 (0.09)	1.03 (0.10)	1.01 (0.10)	1 (0.09)	1.05 (0.21)	1 (0.06)

### EEWMA-MA control chart verses^25^

Here, we investigated the advantages of the proposed charting structure by combining it with Ref.^25^ with the help of ARL. The suggested chart is converted to Ref.^25^ if we put ${\lambda }_{2}=0.$ Table 9 displays the ARL values obtained from Ref.^25^. We observe the lower values of ARL for the offered chart, demonstrating the outstanding execution of the presented charting structure. For example, when ${ARL}_{0}=370,{\lambda }_{1}=0.1$, ${\lambda }_{2}=0.03 c=0.03$ the ARL for the recommended chart is 270.76 and it was 286.34 for the competitor chart. Similarly, for ${\lambda }_{1}=0.2,{\lambda }_{2}=0.07 {\text{ and }} c=0.06$ the value of ARL for the suggested chart is 182.23 and for an opponent chart, it was 207.12. As a result, the best assessment of the proposed chart is shown in the form of lower ARL.

### Proposed *EEWMA-RE* control chart verses^9^

The proposed *EEWMA-RE* chart is converted to Ref.^9^ if we set $\rho =0.0$. We placed the ARL values using^9^ in Table 10. We notice that the execution of the presented charting scheme is remarkable for every value of the shifted constant. For example, when ${ARL}_{0}=370,{\rho =0.90, \lambda }_{1}=0.1$, ${\lambda }_{2}=0.03 c=0.04$ the ARL value for an offered chart is 100.34 and it was 251.12. Similarly, for $c=0.07$, the ARL for the presented chart is 42.34 and for the opponent chart the ARL was 150.32.

**Table 10:** Comparisons of the proposed EEWMA-RE control chart versus existing charts when ${ARL}_{0}=370, n=5$.

	$\rho =0.90$		$\rho =0.0$		$\rho =0.90$		$\rho =0.90$	
	Proposed EEWMA-RE		^9^		^33^		^44^	
	${\lambda }_{1}=0.1,{\lambda }_{2}=0.03$		${\lambda }_{1}=0.1,{\lambda }_{2}=0.03$		$\lambda =0.10$		$\lambda =0.10$	
	$k=2.7194$		$k=2.7194$		$k=2.7180$		$k=2.6430$	
c	$ARL$	$SDRL$	$ARL$	$SDRL$	$ARL$	$SDRL$	$ARL$	$SDRL$
0	372.23	371.41	372.45	373.43	371.12	376.23	370.99	379.11
0.03	150.12	144.98	287.41	290.12	171.12	167.12	164.95	162.52
0.04	100.34	94.21	251.12	248.12	116.32	114.43	110.08	114.01
0.05	73.21	67.98	214.34	215.67	83.23	79.32	80.81	80.95
0.06	54.23	47.32	178.34	175.34	61.23	56.12	59.93	55.35
0.07	42.34	36.12	150.32	149.34	48.12	41.23	44.96	40.12
0.08	33.23	27.12	124.34	116.34	37.43	33.43	36.62	30.87
0.09	27.12	21.23	107.45	101.61	30.32	24.43	29.67	24.94
0.10	21.12	17.23	91.23	87.12	26.32	20.41	24.67	19.86
0.12	16.78	11.23	67.12	65.78	18.32	14.23	17.59	13.86
0.15	10.89	5.23	48.21	49.12	12.23	11.78	11.58	8.27
1.00	1	0	2.11	1.91	1	0	1	0
2.00	1	0	1.03	0.10	1	0	1	0

### Proposed *EEWMA-RE* control chart verses Ref.^33^

In this section, we compare the capability of the proposed *EEWMA-RE* control chart with the chart suggested by Ref.^33^. The suggested chart is transformed to the auxiliary information based EWMA chart if we set ${\lambda }_{2}=0.0.$ The ARL values using Ref.^33^ when $\rho =0.90$ and $\lambda =0.10$ are placed in Table 10. From these results, we see the execution of the offered chart is marvelous in searching out the smaller shifts in process parameters. For example when ${ARL}_{0}=370,{\rho =0.90, \lambda }_{1}=0.1$, ${\lambda }_{2}=0.03 c=0.03$ the ARL value for the presented charting structure is 150.12 and it was 171.12 for Ref.^33^. Similarly, for $c=0.04$, the ARL for the presented chart is 100.34 and for the opponent chart, the ARL was 1116.32.

### Proposed *EEWMA-RE* control chart verses Ref.^44^

Here, the supremacy of the recommended chart is compared with the chart suggested by Ref.^44^. To see a clear picture of the proposed and component chart we take $\rho =0.90$ for both charts for calculating the values of ARL. The ARL values using Ref.^44^ are placed in Table 10. We can see the smaller values of ARL for the proposed chart structure which shows the better performance of the proposed idea. For example, the value of ARL for the recommended chart is 150.12 when ${ARL}_{0}=370,{\rho =0.90, \lambda }_{1}=0.1$, ${\lambda }_{2}=0.03 c=0.03$ and for the competitor chart, it was 164.95.

### Proposed *EEWMA-REMA* and *EWMA-REMA* control charts verses Ref.^45^

In this section, the two suggested control charts (EEWMA-REMA and EWMA-REMA) charts are compared with the chart recommended by Ref.^45^ using the same values of MA span and auxiliary information that is $z=5$, $\rho =0.5$. The first proposed EEWMA-REMA control chart is converted to second EWMA-REMA proposed chart if we set ${\lambda }_{2}=0.0$ and EWMA-REMA control chart is transformed to Ref.^45^ If we set $\lambda =1.$ The ARL values using Ref.^45^ are placed in Table 11. From the results, we see that both proposed charts are much better at searching out the minor variation in process parameters as compared to the opponent chart. For example when ${ARL}_{0}=370,{\rho =0.50, \lambda }_{1}=0.1$, ${\lambda }_{2}=0.03 c=0.04$ the ARL value for the EEWMA-REMA chart is 218.23, for EWMA-REMA chart the value of the ARL is 231.34 when $\lambda =0.10$ and for Ref.^45^ it was 301.36. As a result, the recommended control charts are shown to be more effective in detecting tiny changes.

**Table 11:** Comparisons of the proposed EEWMA-REMA and EWMA-REMA control charts using auxiliary information versus^45^ when ${ARL}_{0}=370, n=5$.

	$$\rho =0.50 {\text{ and }} z=5$$					
	Proposed *EEWMA-REMA*		Proposed * EEWMA-REMA*		^45^	
	${\lambda }_{1}=0.1,{\lambda }_{2}=0.03$		$\lambda =0.10$		$\lambda =1.0$	
	$k=5.018$		$k=5.1754$		$k=2.9588$	
c	$ARL$	$SDRL$	$ARL$	$SDRL$	$ARL$	$SDRL$
0	370.86	388.02	370.08	391.81	371.55	450.83
0.03	269.76	282.97	274.25	290.34	339.92	417.36
0.04	218.23	221.23	231.34	251.43	301.36	364.12
0.05	176.12	184.12	195.34	204.54	275.55	334.25
0.06	145.12	148.13	157.45	165.34	248.64	311.96
0.07	118.23	120.32	127.23	128.87	215.63	260.21
0.08	96.12	94.23	108.12	111.23	187.43	233.88
0.09	80.12	78.32	90.89	89.45	165.41	203.27
0.10	70.12	68.89	75.95	73.28	148.05	182.84
0.12	50.14	46.21	56.12	55.65	112.16	136.46
0.15	33.66	29.16	37.34	35.54	75.46	92.08
0.17	27.44	23.48	29.41	26.18	59.65	75.72
0.20	20.94	16.83	21.75	18.83	40.61	51.15
0.22	17.67	13.78	18.65	15.51	33.02	41.25
0.25	14.46	11.03	14.76	12.03	25.64	31.60
1.00	1.53	0.83	1.55	0.82	2.03	2.12
2.00	1	0.0	1.0	0.0	1	0

### Proposed *EEWMA-REMA* control charts verses Ref.^47^

The proposed EEWMA-REMA control chart is compared with the AIB control chart by Ref.^47^. Setting in-control ARL at 200, the control coefficient value of $L=2.807$ is calculated by Ref.^47^. The same value of the control coefficient is used here. The control constant $k$ for the proposed *EEWMA-REMA* control chart is also calculated for the in-control ARL of 200, ${\lambda }_{1}=0.1$ and ${\lambda }_{2}=0.03$. Results are presented in Table 12. It is clear from the table that *EEWMA-REMA* control chart performs better in detecting out-of-control shifts than Ref.^47^.

**Table 12:** Comparisons of the proposed EEWMA-REMA control chart ($n=5, w=5$) versus Ref.^47^ when ${ARL}_{0}=200$.

		EEWMA-REMA	Abbas et al.^47^	EEWMA-REMA	Abbas et al.^47^
Shift	${\rho }_{yx}$	0.5		0.9	
	$k$	4.525	2.807	4.525	2.807
− 3.00		1.000	1.343	1.000	1.000
− 2.50		1.000	1.881	1.000	1.002
− 2.00		1.002	3.232	1.000	1.039
− 1.50		1.041	7.082	1.000	1.357
− 1.00		1.421	20.296	1.001	3.289
− 0.75		2.093	38.097	1.041	7.212
− 0.50		3.950	75.523	1.434	20.618
− 0.25		11.763	145.415	3.983	76.220
0.00		200.074	200.000	200.342	200.000
0.25		11.870	145.415	3.971	76.220
0.50		3.921	75.523	1.432	20.618
0.75		2.093	38.097	1.044	7.212
1.00		1.422	20.296	1.001	3.289
1.50		1.039	7.082	1.000	1.357
2.00		1.001	3.232	1.000	1.039
2.50		1.000	1.881	1.000	1.002
3.00		1.000	1.343	1.000	1.000

## An illustrated example

**Figure 1:**
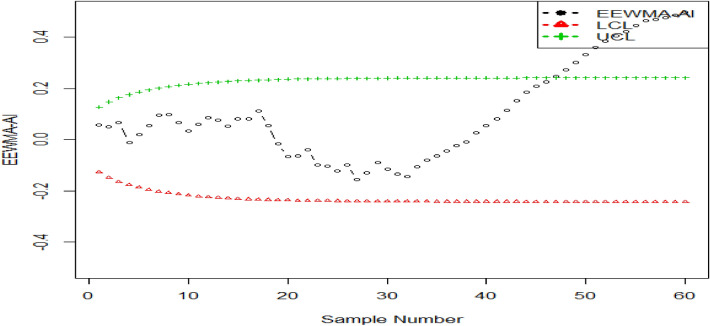
The suggested EEWMA-RE control chart for simulated data.

**Figure 2:**
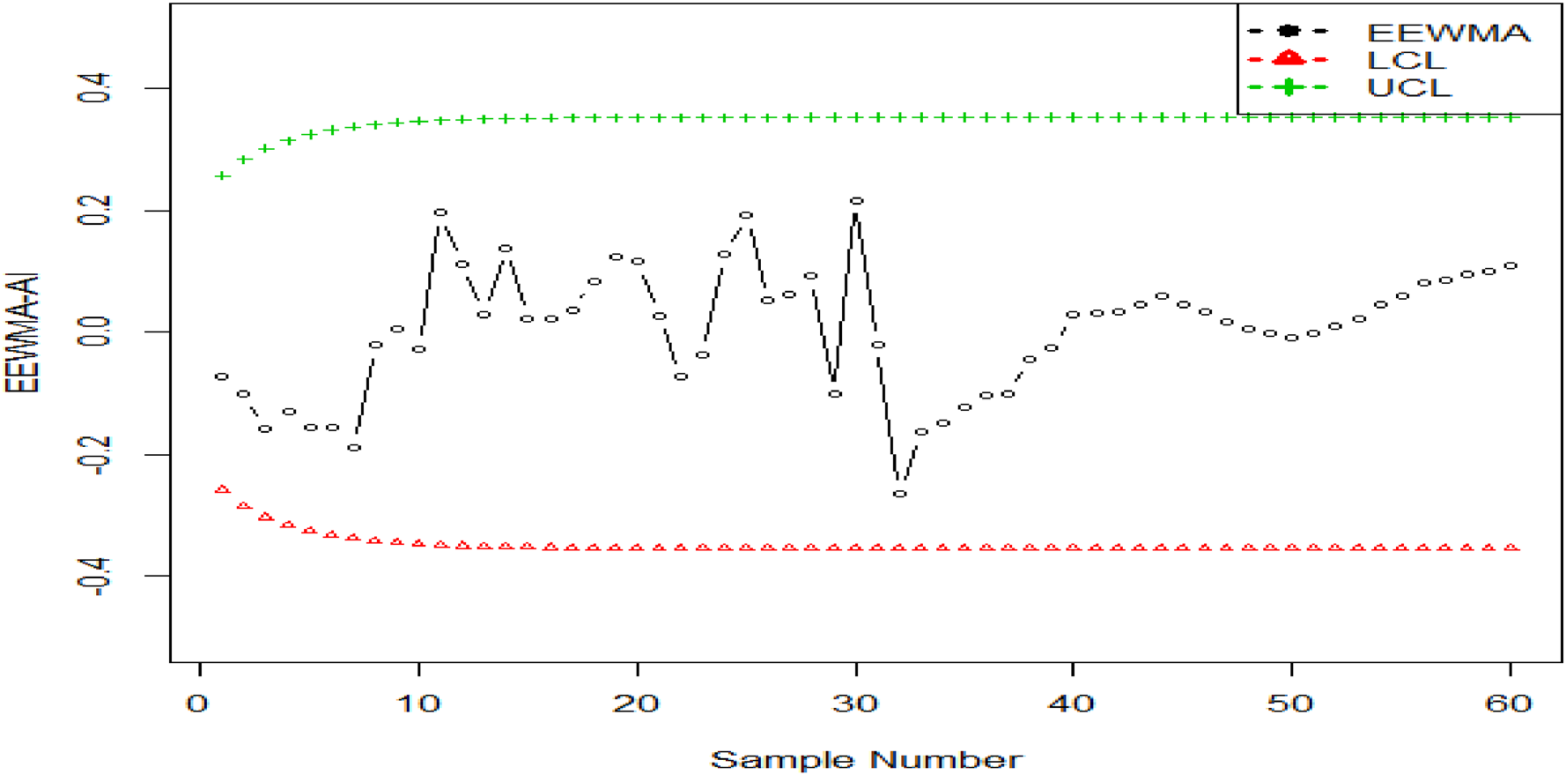
The EEWMA control chart for simulated data.

Here, in this section, we check the competency of the proposed charts by using the simulated data. To save time and space we take one of the proposed charts (say EEWMA-RE control chart) and check its competency using a simulation study. We simulate data using bivariate normal distribution using zero means and unit variances for both variables ($w$ and $y$) using ${\rho }_{wy}=0.90$. The first 30 observations are generated considering the in-control process. The next 30 values are obtained using a shift in the study variable say $c=0.10$. The simulated data is transformed to EEWMA-RE, and the lower and upper control limits (LCL and UCL) are determined using ${\text{ARL}}_{0}=370,{\text{ n}}=5,{\lambda }_{1}=0.1, {\lambda }_{2}=0.03, {\text{n}}=5,{\text{ k}}=2.7194$ and $\rho =0.90$. The presented EEWMA-RE control chart is plotted in Fig. 1. It is seen that an out-of-control signal is generated at the 46-th observation for the EEWMA-RE control chart (the same value is observed in Table 2). We also simulated 30 observations for the in-control process using a standard normal distribution, and another 30 observations for the shifted process using a shifted value in the process mean say $c=0.10$. After getting the simulated data we convert it to the EEWMA statistic and calculate its LCL and UCL using ${\text{ARL}}_{0}=370,{\text{ n}}=5,{\lambda }_{1}=0.1, {\lambda }_{2}=0.03, {\text{n}}=5$ and ${\text{k}}=2.7194$. The EEWMA based simulated data is shown in Fig. 2. From Fig. 2, we see that no out-of-control signal is triggered^9^.

## Industrial application

**Figure 3:**
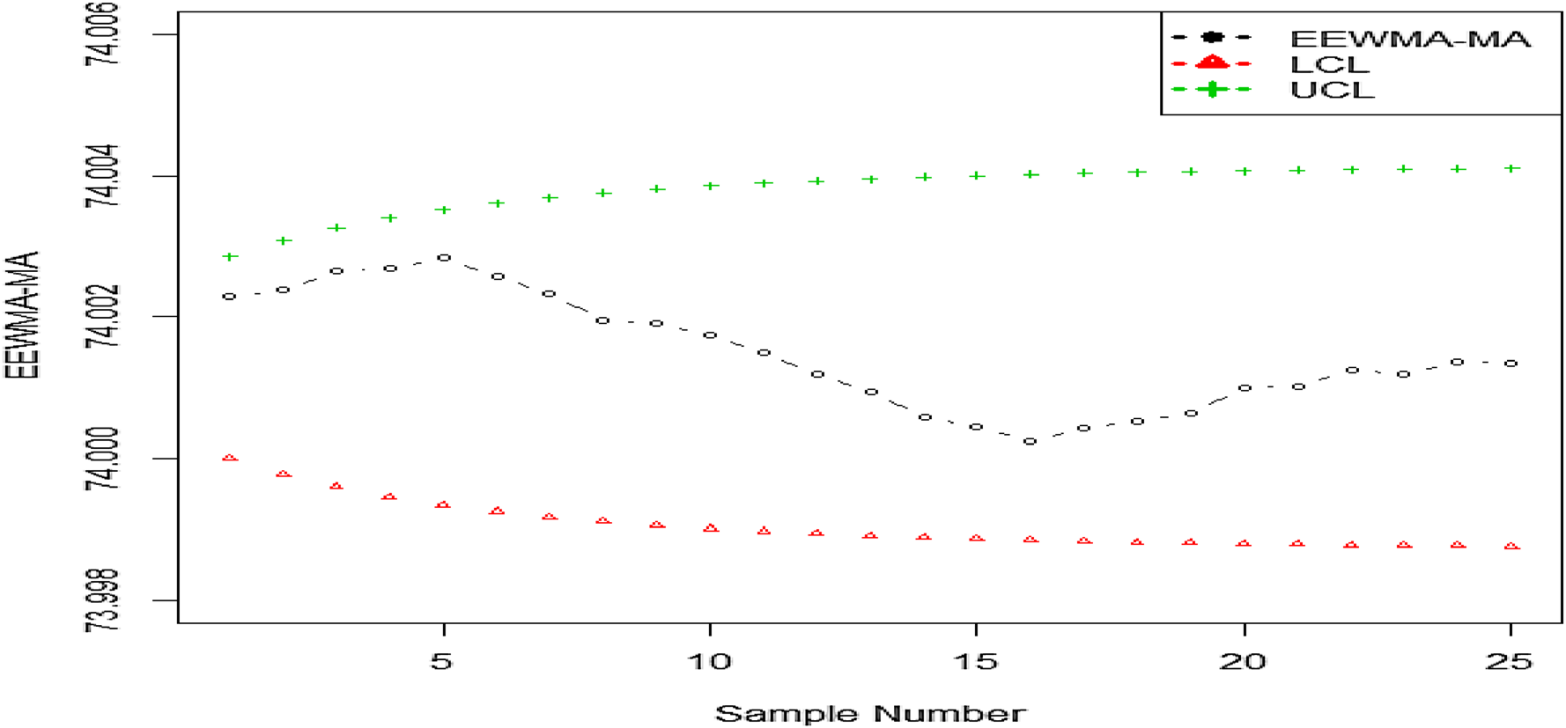
Graph of real-life data of proposed EEWMA-MA chart.

In this section, we analyze the effectiveness of the proposed idea utilizing real data about internal diameter measurements (in mm) for automobile engine piston rings. The data is taken from Ref.^3^. This data set is also used by Ref.^50^. The given data set is based on a subgroup of size five. We calculated the mean value of each subgroup denoted by $\overline{Y }$ and put it in Table 13. After getting the value of $\overline{Y },$ we calculated the value of MA statistic using $z=3$ as ${MA}_{1}=$ 74.010, ${MA}_{2}=\frac{74.010+74.001}{2}=74.0055, {MA}_{3}=\frac{74.010+74.001+74.008}{3}=74.0060$ and so on. Finally, the values of the proposed statistic *EEWMA-MA*_*j*_ using ${\lambda }_{1}=0.1$ and ${\lambda }_{2}=0.03$ as *EEWMA-MA*_*1*_ = ${MA}_{1}-{\lambda }_{2}{MA}_{0}+\left(1-{\lambda }_{1}+{\lambda }_{2}\right){MA}_{0}$, where ${MA}_{0}$ is the overall mean of ${MA}_{j}$. *EEWMA-MA*_*2*_ = ${\lambda }_{1} {MA}_{2}-{\lambda }_{2}{MA}_{1}+\left(1-{\lambda }_{1}+{\lambda }_{2}\right){EEWMA{\text{-}}MA}_{1}$ and so on. The calculated values of the MA statistic and proposed *EEWMA-MA*_*j*_ statistic are shown in Table 13. We calculate the lower and upper control limits of *EEWMA-MA*_*j*_ based proposed statistic using ${ARL}_{0}=370, n=5, z=3$ and $k=4.1550$. The plotting statistic along with LCL and UCL are shown in Fig. 3. From Fig. 3, we see that the process is in-control. However, the 5-th value of plotting statistic is very nearer to the UCL, which could cause an alarming situation for quality personals but still the process is in control and the next values are also in-control. So, no out-of-control signal is generated.

**Table 13:** The values of statistic of inside diameter measurements.

$\overline{Y }$	MA statistic using $z=3$	EEWMA− MA	LCL	UCL
74.0100	74.0100	74.0023	74.0000	74.0029
74.0010	74.0055	74.0024	73.9998	74.0031
74.0080	74.0060	74.0027	73.9996	74.0033
74.0030	74.0040	74.0027	73.9995	74.0034
74.0030	74.0047	74.0028	73.9994	74.0035
73.9960	74.0007	74.0026	73.9993	74.0036
74.0000	73.9997	74.0023	73.9992	74.0037
73.9970	73.9977	74.0020	73.9991	74.0038
74.0040	74.0003	74.0019	73.9991	74.0038
73.9980	73.9997	74.0017	73.9990	74.0039
73.9940	73.9987	74.0015	73.9990	74.0039
74.0010	73.9977	74.0012	73.9989	74.0039
73.9980	73.9977	74.0010	73.9989	74.0040
73.9900	73.9963	74.0006	73.9989	74.0040
74.0060	73.9980	74.0005	73.9989	74.0040
73.9970	73.9977	74.0003	73.9989	74.0040
74.0010	74.0013	74.0004	73.9988	74.0040
74.0070	74.0017	74.0005	73.9988	74.0041
73.9980	74.0020	74.0006	73.9988	74.0041
74.0090	74.0047	74.0010	73.9988	74.0041
74.0000	74.0023	74.0010	73.9988	74.0041
74.0020	74.0037	74.0013	73.9988	74.0041
74.0020	74.0013	74.0012	73.9988	74.0041
74.0050	74.0030	74.0014	73.9988	74.0041

## Limitations

In this section, we will discuss the advantages and limitations of the proposed MA and RE control charts using EEWMA statistic. As mentioned earlier, the proposed control charts are flexible and several control charts are the special case of the proposed control charts. The extensive simulation study showed that the proposed control charts perform better than the many competitors’ control charts in terms of ARL and SDRL. The proposed control charts have some limitations in that the evaluation of these MA and RE control charts using EEWMA statistic is not simple to compute as compared to traditional EWMA control. The selection of smoothing constants for MA and RE using EEWMA statistic is not easy and makes the computational work more difficult. Another limitation of the proposed MA and RE control charts using EEWMA statistic is that they can be used only when the variable of interest follows the normal distribution. The RE control chart using EEWMA has the limitation that it can be applied for correlated auxiliary variables.

## Concluding remarks

Control charts are very helpful instruments to observe the process parameter (s). The auxiliary information is mostly used to improve the reliability of the estimation in the survey sampling. By inspiring this reality, several researchers presented auxiliary information -based control charts to improve the competency of the industrial processes. In this study, we have suggested four types of memory-based control charts using with and without auxiliary information to enhance the detecting ability of the process location parameter. The ARL values of the presented charts are computed with different choices of correlation coefficient (${\rho }_{YX}$) and span (z). From the results, it is noted that the performance of the presented charts is improved with an increase in the value of z. It is also observed that the efficiency of the proposed framework also improves as the correlation coefficient between the study variable and auxiliary information becomes elevated. We also acknowledged the excellent assessment of the suggested charts for a lower value of the smoothing constant. The presented charts perform efficiently relative to the existing counterparts incorporated in this study. Proposed control charts are compared with Refs.^47,33,51,44,9,25^. It is found that the proposed control chart performs better in detecting small shifts than all of these control charts. The proposed control chart can be applied in many industries including example automobile industry, service industry and manufacturing industry. The scope of this study can be expanded to observing the dispersion and multivariate structures.

## Data Availability

The data is given in the paper.
